# Exploring Female University Students' Participation in Physical Activity in Saudi Arabia: A Mixed-Methods Study

**DOI:** 10.3389/fpubh.2022.829296

**Published:** 2022-03-18

**Authors:** Najla Aljehani, Husna Razee, Jan Ritchie, Trinidad Valenzuela, Anne Bunde-Birouste, Ghadah Alkhaldi

**Affiliations:** ^1^School of Population Health, The University of New South Wales, Sydney, NSW, Australia; ^2^Department of Public Health, College of Health Sciences, Saudi Electronic University, Riyadh, Saudi Arabia; ^3^Faculty of Medicine and Health, Sydney School of Health Sciences, The University of Sydney, Sydney, NSW, Australia; ^4^Exercise and Rehabilitation Sciences Laboratory, Faculty of Rehabilitation Sciences, School of Physical Therapy, Universidad Andres Bello, Santiago, Chile; ^5^College of Applied Medical Sciences, King Saud University, Riyadh, Saudi Arabia

**Keywords:** women's physical activity, Saudi Arabia, physical activity barriers and motivators, socioecological framework, cultural norms in Saudi Arabia

## Abstract

**Background:**

The importance of physical activity in improving physical and mental health has been emphasized in many studies. Researchers in Saudi Arabia have reported an increase in physical inactivity among Saudis, especially among University female population. Current efforts in the field in Saudi Arabia have yet to explore barriers and facilitators that influence female University student's participation in physical activity. This study aims to provide an assessment of the situation regarding physical activity among female University students in Saudi Arabia.

**Methods:**

This mixed method study preceded a participatory action research initiative. The first part of the mixed method consisted of a cross-sectional survey of 375 female University students in Saudi Arabia who completed the short form of the International Physical Activity Questionnaire. The second part consisted of semi-structured, in-depth interviews with 14 female University students and 16 female exercise trainers.

**Results:**

Results showed that most participants (91%) spent more time in walking activity compared to moderate (66%) and vigorous activity (57%) for at least 10 min at a time over a period of 7 days. Results showed that 70% of participants did not meet the WHO recommendation of 150 min per week of moderate activity, while around 62% of participants did not meet the WHO recommendation of 75 min per week of vigorous activity. Barriers to participation included limited facilities for physical activities, academic workload, gender role, and the need to adhere to cultural standards. Facilitators included valuing positive results, general health concerns, and family support.

**Conclusion:**

Knowledge gained from this study might support organizations and public health authorities to develop physical activity interventions that better address Saudi women's perceived needs. These findings are an important contribution to current knowledge in light of recent advances in women's rights in Saudi Arabia.

## Introduction

Women's engagement in sports and physical activities has a long history of discrimination and division, impacted by a variety of social and cultural conventions and beliefs (1). Globally women encounter barriers in accessing sports and participating in physical activities (2–4). Consequently, women's opportunities for an active lifestyle are affected at disproportionately higher levels than for men and therefore lead to increased inactivity levels for women (5). This gender disparity was highlighted in the 2011 World Health Organization (WHO) report on non-communicable diseases (NCDs), which clearly showed that men are more physically active than women in all WHO regions (6). According to the WHO, physical activity has broad, long-term health benefits for individuals (7) and inactivity is ranked as the fourth leading risk factor for mortality (6). Physical activity is proven to reduce the risk of NCDs such as stroke, diabetic complications, cancer, and coronary heart disease (7–9).

Research into women's physical activity in Saudi Arabia has consistently shown a high prevalence of inactivity (10). For instance, Alhakbany, Alzamil (11) reported 50% level of inactivity among adult Saudi females. This poses a challenge as physical inactivity has detrimental effects on Saudi women's health (12, 13). An example is obesity, as Al-Quwaidhi, Pearce (14) indicated that 68% of Saudi women are obese and this percentage was expected to reach 78% in 2020 (15). Barriers to women's participation reported in previous studies included being time-poor owing to competing responsibilities, scarcity and high cost of sports facilities, and lack of motivation (16, 17). However, much of the research undertaken to date has used quantitative approaches to measuring physical activity levels and identifying barriers and facilitators to physical activity participation (18). Such approaches are limited in obtaining a deeper understanding of Saudi Arabian women's experiences with physical activity. Yet a deep understanding is essential to plan and implement effective public health strategies for increasing physical activity levels among Saudi women.

In Saudi Arabia, women's views toward physical activity have been heavily influenced by societal and cultural norms (1). Saudi society tends to be a male-dominated collective society, and its conservative norms have defined traditional rules for women (1). As a result, women's physical activity needs have long been overlooked, and women in the country face numerous barriers to participating in physical activity. For example, it is generally accepted for men to be active in public spheres of life, be it for work, sports, or social activities. However, women face cultural and social pressures that reduce their opportunities for being actively engaged in the public sphere (19). Also, women did not receive physical education or participate in sports in schools or universities until 2019 and they were not taught about the importance of physical activity for health. This has contributed to community expectations and norms that restrict women from engaging in physical activity in public (20, 21). Moreover, women's physical activity and sports receive substantially less funding and support than men's facilities and teams (22) which perpetuates the existing cultural norms and adds a further structural barrier for women's physical activity.

Recently, however, as part of the Saudi Vision 2030 social policy statement (23), the Saudi government has seen benefits in investing in population health and focusing on increasing physical activity levels in its population, especially among females (24). As a sign of their commitment to improving women's physical activity, the Kingdom has created a new department for women under the Kingdom's General Authority for Sports. This is an important step to promote greater access to sports for women. However, for real change in activity levels to occur, it is necessary to have evidence that supports not just the design of interventions, but the development of effective policies and practices. This would necessitate a deeper understanding of Saudi women's lived experiences, as well as research into the extent to which cultural norms and practices influence their physical activity participation (25). This article reports on research that was undertaken as part of a larger participatory action research study that looked at identifying approaches for improving physical activity levels among female University students in Saudi Arabia.

University students are an interesting population group to study as universities are likely to provide environments that encourage efforts to promote health through educational and environmental influences (18, 26). The transition from high school to University is one of the most significant changes in an individual's life (27). Studies have shown that University students are at a higher risk of weight gain than the general population by 5.5 times (28). Studies also showed that physical activity and exercise are not only helpful for reducing the psychological stress that University students can encounter, but they also promotes mood, self-esteem, physical and mental health (29, 30). In Saudi Arabia, studies among University students have reported low levels of physical activity especially among female students, mainly due to time constraints, highlighting the need to increase physical activity to reduce stress and improve health (10, 26, 31).

Studies that draw on experiences of University students provide an evidence-based foundation for universities in Saudi Arabia to develop their health promotion strategies. Moreover, University students constitute a significant proportion of the Saudi population as young people make up the majority of the Saudi population (3,920,060 young females from around 14 million females in Saudi Arabia) (32). University students are also considered a group that are easily accessible and can provide rich thick data. Therefore, our study focused on female University students with the aim to (a) assess female University student's physical activity level; and (b) explore the barriers and facilitators that influence their participation in physical activity. This study was guided by the following research questions:

1. What are the current levels of physical activity among female students attending King Saud University (KSU)?
2. What are the barriers and facilitators for female University students to participate in physical activity?

## Methods

### Design

This article reports on the first part of a larger participatory action research study. This phase of the study employed a convergent parallel mixed method approach (33) using both quantitative and qualitative techniques including a cross-sectional survey and in-depth interviews.

### Study Setting

The research took place on the female-only campus of KSU in Riyadh, Saudi Arabia's capital. KSU was Saudi Arabia's first and now a main public university, established in 1957. The diversity of students drawn from across the country enabled us to explore the experiences of females from different social and economic backgrounds and enabled some degree of variation in perspectives. While the student population includes females from varying socioeconomic backgrounds since no tuition fees are charged, given that only 20% of the female population in Saudi Arabia attend universities (34), it must be emphasized that University students are privileged in many ways compared to those who do not attend universities. Thus, even though KSU is a public university, it may not be representative of the Saudi female population.

### Data Collection

#### Quantitative Data

##### Prevalence of Physical Activity Among KSU Female Students

An on-line based survey using the validated Arabic short version of the International Physical Activity Questionnaire (IPAQ) was used to assess the current physical activity levels among KSU female students (17, 35–37). The survey aimed to determine the amount of physical activity in which adults aged 15 to 69 engaged in the 7 days before taking the survey as a population surveillance measure (37). Data from the IPAQ included time spent in walking, moderate and vigorous-intensity activity, and sedentary activity. Data for the age, marital status (single, married, widowed, divorced), height and weight of participants were also collected. Height and weight were used to calculate participant's Body Mass Index (BMI). Participants' weekly recorded minutes of low, moderate, and high-intensity physical activity were used to classify their physical activity level in relation to the WHO recommendations of at least 150 min per week of moderate-intensity physical activity, or at least 75 min per week of vigorous-intensity physical activity (38). For additional health benefits, the WHO recommends adults engage in 300 min of moderate-intensity activities, or 150 min of vigorous-intensity activities, or an equivalent combination of both per week (38).

The survey participants were recruited using a purposive sampling approach (39). An invitation to participate in the study was sent via WhatsApp to groups of registered female students in the application. A study by Alsanie (40), indicates that around 93% of Saudi Arabia's University students use the WhatsApp application, hence the reason for choosing this approach to participant recruitment.

The sample size for this study was determined using Jaykaran and Tamoghna (41) formula for survey sample size calculation. In 2016, KSU had ~28,917 female students (42). With prior research estimating the prevalence of inactivity to be around 44% (18), 378 individuals were needed for the survey to achieve a 95% confidence interval and a 5% significance level.

#### Qualitative Data

##### In-depth Interviews to Explore Barriers and Facilitators to Engaging in Physical Activity

To gain a better understanding of the barriers and facilitators to women's participation in physical activity in Saudi Arabia, in-depth interviews with female students and female exercise trainers were conducted. Inclusion of trainers helped obtain alternative perspectives on Saudi Arabian women's physical activity and to triangulate data sources, thus increasing the credibility and trustworthiness of the study (43). A semi-structured interview guide was used in these interviews. The interview guide was developed by reviewing the relevant literature in the field with all authors contributing. In the interviews with students, the focus was on their physical activity experiences and their perceived barriers and facilitators to being physically active. Interviews with female exercise trainers explored their views about the physical activity status of women in Saudi Arabia and their experiences with women engaging in physical activity. Interviews were conducted by NJ, a Saudi female native Arabic speaker with a MD in Public Health who was also a PhD candidate during the period of data collection. All interviews were audio recorded. Each interview lasted ~1 h. Pseudonyms are used for students and trainers. NJ transcribed all the interviews. After the first interview, NJ and GK listened to the interview to identify areas where the interviewing skills could be improved. A research journal was maintained during the data collection, one component of an audit trail meeting the rigor criterion of dependability (44, 45). The journal enabled the tracking of the research process and recorded any changes made to the original design, following the flexibility that a qualitative approach provides (44). Venues for the interviews were determined based on interviewee preference. All students preferred interviews conducted at the University during their break times. Of the key informants, six preferred to be interviewed at the gym where they worked; KSU was a convenient place for another two; and the remaining eight interviews took place in public places, mainly cafés.

Students were invited to participate in the interview at the end of the survey and asked for their contact information if they wished to do so. Snowball sampling was also utilized to recruit female exercise trainers and students, with those who agreed to participate being asked to identify someone else who might be interested (46).

Following the advice of Guest and Bunce (47) who indicated that data saturation is likely to occur after 12 interviews, a minimum of 12 interviews with female students at KSU and 12 interviews with female trainers were planned. However, data was collected until the researchers were satisfied there was enough data to answer the research questions and develop meaningful themes.

## Inclusion Criteria

Students had to meet the following inclusion criteria to be eligible to participate in both the survey and the interviews: (i) not be pregnant at the time of recruitment, (ii) not have had bone fractures within the past 6 months, or injuries that affected their ability to perform any type of physical activity; and (iii) not have an unstable or acute medical condition that precluded participation in physical activity.

Female exercise trainers were eligible if they had at least 6 months' experience as an exercise trainer in Saudi Arabia to ensure sufficient exposure to the issues being researched and hence, their ability to provide rich data about the topic.

## Data Analysis

### IPAQ Survey

SPSS version 25 was used to analyse the data. Participant characteristics, including age, marital status, and body mass index (BMI) were reported using descriptive statistics. IPAQ data were processed and scored following established guidelines for data processing (37).

### Interviews

Interviews were transcribed verbatim and thematically analyzed following an inductive approach guided by Braun and Clarke's six-step process (48). As the interviews were undertaken in Arabic, translation was required into English. A back translation process was followed to ensure the quality of the translation process and ascertain that the meaning was not lost (49). After all the interviews were translated into English, the English transcripts were uploaded to NVivo for data coding and management. Two of the authors (NJ and GK) coded two interviews separately and then compared the coding. Through this process a coding manual with code descriptions was developed and used for further coding. As coding progressed this was further revised through consensus discussion between NJ & GK. In this way, shared interpretation benefitted the credibility of the findings. Then, at the stage of developing themes, there were discussions between the authors, and a consensus approach was used to finalize the themes.

## Results

Data collection for the survey took place between April and May 2018. A total of 1,142 responses were received of which 375 valid responses were used for analyses. Reasons for excluding responses included missing data (*n* = 752) and responses where the total sum of walking, moderate and vigorous time variables was >960 min, as these were considered outliers (*n* = 15) (48). The mean age of the survey participants was 19.9 years (range 18–28 years; SD = 1.7). Among participants, 88% were single. More than half of the participants (58%) were of normal weight, while 20, 12, and 10% were found to be overweight, obese, and underweight, respectively.

Following the survey, a total of 14 interviews with female students at KSU and 16 interviews with female exercise trainers were conducted over 2 months between May and June 2018. Twelve of the female exercise trainers worked at gyms while four did not work in a gym but had their own private business where they provided personal training, online consultations, and nutritional programs. Tables 1, 2 provide a brief background on the participants who were interviewed, both students and trainers.

**Table 1:** Brief background on KSU students who were interviewed.

**Name**	**Particulars**
Noura	19 years old. Second year student, specializing in Business Administration
Maryam	19 years old. Second year student, specializing in Law and Political Science.
Nuha	20 years old. Second year student, specializing in History Science.
Fatimah	19 years old. Second year student, specializing in Social Science.
Noor	23 years old. Fifth year student, specializing in Botany and Microbiology Sciences.
Dana	20 years old. Second year student, specializing in Laboratory Science
Sarah	19 years old. First year student, specializing in linguistics
Duaa	19 years old. Second year student, specializing in Business Administration
Ebtisam	20 years old. Third year student, specializing in Dentistry Science
Ghadah	21 years old. Third year student, specializing in nutrition science
Seba	22 years old. Fourth year student, specializing in mathematical science
Mashel	20 years old. Second year student, specializing in Optometry Sciences
Hala	21 years old. Third year student, specializing in social science
Esraa	20 years old. Second year student, specializing in Optometry Science

**Table 2:** Brief background on female trainers who were interviewed.

**Name**	**Particulars**
Dalal	Saudi trainer with 5 years of experience. Works in a gym with expensive membership.
Asma	Saudi trainer with 7 years of experience. Has her own private training business. Provides personal training, online consultations and training and nutrition programs.
Samyah	Syrian trainer with almost 2 years of experience. Works in a gym with an average membership.
Majedah	Saudi trainer with 2 years of experience. Has her own private training business. Provides personal training, online consultations and training and nutrition programs.
Rawan	Saudi trainer with 2 years of experience. Used to work in a gym with an average membership, then recently moved to work at KSU gym.
Ghadeer	Saudi trainer with 3 years of experience. Works in a gym with expensive membership.
Waad	Saudi trainer with 3 years of experience. Works in a gym with an average membership.
Amal	Jordanian trainer with 10 years of experience in Saudi Arabia. Works in a gym with an average membership.
Heba	Saudi trainer with almost 2 years of experience. Works in a gym with an average membership.
Rasha	Tunisian trainer with 5 years of experience working in Saudi Arabia. Had a bachelor's degree in physical fitness from Tunis. Works in a gym with an average membership.
Alaa	Saudi trainer with 6 months of experience. Has her own private training business. Provides personal training, online consultations and training and nutrition programs.
Lamya	Saudi trainer with 8 months of experience. Has her own private training business. Provides personal training, online consultations and programs and group workout sessions in a rented studio.
Afnan	Saudi trainer with 10 years of experience. Works in a gym with expensive membership.
Eman	Saudi trainer with 7 years of experience. Works in a gym with expensive membership.
Maha	Saudi trainer with 10 years of experience. Used to work in a gym with expensive membership, then recently moved to a hospital gym.
Sultanah	Saudi trainer with 1 year of experience. Works in a gym with expensive membership.

### Physical Activity Level

According to the IPAQ's categorical scoring protocol (37), 43% of participants were highly active, 28% were moderately active, and 29% reported participating in low levels of physical activity. The median and interquartile ranges for the total minutes per week and the total Metabolic Equivalent of Task (MET) per minute per week of the three physical activity domains and total physical activity across all domains are listed below in Table 3.

**Table 3:** Median and interquartile ranges for participants who reported engaging in any physical activity.

	**Total MET-min/WK Median (IQR)**	**Total min/WK Median (IQR)**
All domains of PA	2,034 (3,529)	120 (160)
Vigorous PA	240 (1,440)	30 (180)
Moderate PA	240 (720)	60 (180)
Walking PA	693 (1,551)	210 (470)

*IQR, interquartile range; MET-min/WK, metabolic equivalent minutes per week*.

Results showed that most participants (91%) spent more time in walking activity compared to moderate (66%) and vigorous activity (57%) for at least 10 min at a time over the past 7 days. Table 4 indicates the percentage of female students who walked or exercised at moderate or vigorous intensities. At the bottom of the table is the proportion of participants (mean SD) who engaged in at least 10 min of walking, moderate, or vigorous physical activity in the previous 7 days.

**Table 4:** Proportions (%) of participants who engaged in walking, moderate, and vigorous physical activity over the past 7 days.

**Number of minutes per week**	**Walking**		**Moderate activity**		**Vigorous Activity**	
	**Frequency**	**Percentage (%)**	**Frequency**	**Percentage (%)**	**Frequency**	**Percentage (%)**
0	35	9.3	126	33.6	162	43.2
10–30	31	8.3	35	9.3	38	10.1
31–60	27	7.2	43	11.5	31	8.3
61–149	55	14.7	60	16	40	10.7
150–299	72	19.2	41	11.2	45	12
300 or more	155	41.3	70	18.4	59	15.7
	N (%)	mean ± SD	N (%)	mean ± SD	N (%)	mean ± SD
Total^*^	340 (90.7%)	(315 ± 372)	248 (66%)	(179 ± 300)	213 (57%)	(129 ± 218)

* *Totals are based on the number of participants who engaged in walking, moderate, and vigorous, activities for 10 min or more per week as per the IPAQ scoring protocol (37)*.

The findings showed that 30% of participants met the WHO recommendation of 150 min per week of moderate physical activity. Similarly, 38% of participants met the recommendation of 75 min per week of vigorous physical activity. Only 18 and 28% of female students were achieving the WHO recommendations for additional health benefits for moderate and vigorous categories, respectively (Table 5).

**Table 5:** Proportion of participants achieving WHO recommendations for moderate and vigorous physical activity.

**Minutes per week**	**Frequency**	**Percentage (%)**
**WHO recommendations for moderate activity**		
149 or less	264	70.4
150–299	42	11.2
300 or more	69	18.4
Total	375	100
**WHO recommendations for vigorous activity**		
74 or less	231	61.6
75–149	40	10.7
150 or more	104	27.7
Total	375	100

### Barriers to Participation in Physical Activity

Three main themes were identified regarding barriers to participation in physical activities. Key themes were environmental and structural factors restricting engagement with physical activity, personal factors which made physical activity a lower priority, and social and cultural norms and expectations which negatively influenced participation. Table 6 provides a summary of the main themes and subthemes.

**Table 6:** Summary of the main themes and the subthemes from the in-depth interviews.

	**Themes**	**Subthemes**
Barriers to participation in physical activity	Unsuitability of environment Difficulties in accessing facilities	Extreme climate conditions Road's design and safety concerns Limited female facilities High gym fees
	Personal barriers	Academic workload Not seeing physical activity as a priority
	Social contextual factors	Soia-cultural expectations regarding a socially acceptable body image Restrictive gender role Pressure to adhere to cultural and religious standards.
Motivators to participation in physical activity	Personal motivators	Noticing positive results from physical activity Having a company to workout with General health concerns Having a high level of self-determination
	Family support	Mothers as the main source of support Being influenced by active family members

#### Environmental and Structural Factors Restricted Engaging With Physical Activity

This theme reflected barriers over which participants had minimal control, including the suitability of the environment, and difficulties found in accessing facilities for undertaking physical activities. Participants clearly recognized Saudi Arabia's climate—extremely hot in summer and cold in winter—as a deterrent to physical activity. Mashael, a student participant, confirmed this view by saying, “The weather here in Riyadh is a serious problem.” In Saudi Arabia, women must wear an *abaya*, a full-length outer garment worn by Muslim women outside of their homes. Wearing the abaya in summer made it challenging for participants to engage in outdoor activities. However, in the interviews, they cited this as a burden for considering outdoor activities reluctantly, as this may have given the impression that they did not value the dress code. Dana, a student in her 2nd year stated this point and then said that even if she did not wear an abaya, it was hot.

The hot weather is a main barrier, here [in Riyadh] it is too dry and hot, especially when you are wearing the Abaya … even if we [females] are not wearing the Abaya, still too hot to bear (Dana, student).

Lack of footpaths was a barrier participants highlighted when considering walking as a physical activity. In Saudi Arabia, the roads and roadsides are not designed and built with pedestrians in mind. Asma, a Saudi trainer, explained how “the environment is not prepared for [walking], there are no sidewalks,” and because of that “it is not easy to cross the road because cars might hit you.” These issues of the built environment were echoed by students too, who felt “terrified when [they] cross the street” and, in the absence of dedicated paths for cyclists and pedestrians, injuries or fatalities could result.

In Saudi Arabia, there are no mixed-gender sports facilities, and females are only allowed to access female-only gyms. Participants pointed out how the limited facilities for physical activity was a barrier for them. Typical of most participants, Hala, a student noted the scarcity of female-only gyms in Saudi Arabia as a key barrier to being physically active:

Lack of gyms in the area can be discouraging to whoever wants to stay fit and be active. There are few gyms here in Riyadh, which is not motivating for us at all (Hala, student).

Apart from gyms, there are few other options for physical activity. Students spoke of activities such as fencing, marathons, and other types of sport that they would like to try if opportunities were available in Riyadh.

I love fencing, but I never tried it before. One reason for not practicing it is the lack of gyms or trainers specialized in it (Noura, student).

Further, for many of the students, the lack of variety in facilities was also a deterrent to physical activity. However, Lamya, one of the trainers had a different perspective. For her, it wasn't just the availability of facilities, but the lack of awareness of the range of activities that women could do.

Usually, sport for them [females] here is walking, joining the gym, and sometimes swimming, no, there's a lot to do, think out of the box, out of their box (Lamya, trainer).

The limited number of female-only gyms made their fees expensive compared to those at male-only gyms. Thus, for many women, especially students, the exorbitant cost was problematic. Students mainly relied on their families to pay their expenses, and in families with more than one daughter, parents could not spend such a large amount of money on gym fees.

Men's gym subscription fees are reasonable. But for us [females] we must pay around SAR8000-9000 for a year [USD 2132-2400], I even went to a gym, and [they] told me that their fees are SAR10000 per year [USD 2670] (Seba, student).

#### Personal Factors Made Physical Activity a Lower Priority

Personal barriers were factors that limited participants' ability to engage in physical activity for reasons that were personal to their particular situation. Personal barriers included academic workload and not perceiving physical activity as a priority.

For students, the academic workload was a deterrent. According to Ebtisam, a 3rd-year dentistry student, “sport comes in second place for me, studying is more important.” The lengthy daily schedule of classes, homework, and extra-curricular study responsibilities left students feel so exhausted that at the end of the day they “just wanted to sleep.” The significance of academic workload, particularly during exam time was clearly portrayed in Nuha's words: “When it is examinations period, I barely have time to breathe let alone doing exercise.”

Noor, a student in her final year of microbiology, spoke differently about academic load. She believed that everyone could find time to work out if they planned their schedules. For her, students who used studying and University as an excuse did not make sense because, she felt, “they do not spend the 24 h studying … it is not an excuse.” According to Noor, it was a matter of making physical activity a priority; if students were more organized, she believed, “they will find plenty of time to go out for walking or do any sort of exercise.”

Students' responses clearly suggested physical activity was not a priority with academic goals coming first. The choice was not limited to studying vs. exercising; many students preferred to rest or do anything else rather than to exercise in their free time. Fatimah, a social science student, stated, “It seems like I lack time to do sports, but honestly there is time, I just prefer spending it lying down, thinking, and resting.”

#### Social and Cultural Norms and Expectations Negatively Influenced Participation

Social barriers referred to those that arose from the perceptions and expectations of the wider Saudi community about women participating in physical activity, particularly the social and cultural norms around women's physical activity. According to the participant's response, the socio-cultural expectations surrounding women's physical activity that might negatively influence their participation were related to body image, restrictive gender roles, and the pressure to adhere to cultural and religious standards.

It was clear that wanting to have a “perfect” body contributed to young women seeking quick ways to achieve that without having an exercise routine. Both trainers' and student responses suggested there is an overwhelming concern about body image. Noor, a final-year student, had exercised regularly for a long time and talked about how “being in a good shape is the main reason for females to be in the gym.” Trainers indicated body shape was a driver for exercise noting that the reason most women came to the gym was “to lose weight and nothing else,” and most of the time it was because “they had a short-term goal,” such as wanting “to buy an outfit [they] like, or seeking a good shape, but not for a healthy body.” They also talked about how “families do not let [thin females] join the gym” and “refuse to pay for [them] because [they] are skinny,” while the opposite was true of obese women—“their families paid for them.”

Responses from both students and trainers reflected some of the community beliefs around what a male or female body should look like. Some of these beliefs related to how weightlifting would cause women to look like men. Noor's words below, recalling a relative's reaction when she started lifting weights in the gym portrayed community perceptions of how a male or female body should look like.

There was a time when someone has told me, do you lift weights? Are you crazy? And I replied, I mean what is wrong with lifting weights? She answered back saying, no, you are not a man to lift weights. Lifting weights will build muscles for you! (Noor, student).

Trainers' too reflected similar beliefs to what was portrayed by Noor. They spoke of their struggle with this notion of building muscles like men. Dalal, a trainer spoke about how this belief was still common among trainees in the gym where she trained, noticing that some trainees stopped when the muscle mass increased because “they did not want to be muscular.”

The participants explained how the prevailing male dominance in Saudi society restricted women's options to practice physical activity due to two main factors. The overprotection of men toward women was one reason for the limits on women's ability to travel independently without a male relative. Second, was the common belief that sport was the domain of men only.

Until very recently, women were required to have their male guardian's permission to pursue an activity, for instance, to join a gym, or to leave the house. As of 2019, women over the age of 18 are no longer subjected to guardianship laws. However, the students felt that men, primarily fathers, and brothers, were concerned when their wives, daughters, and sisters went out in public, for example, to walk in parks, because they would be vulnerable to harassment by other men. Hala, a student, stated that “people here are afraid of harassment against their female relatives if they went out for a walk in the park.” This made it hard for women to get approval to go out alone to walk in a public place.

Harassment from males was noted by the study participants as a reason for not feeling comfortable going out for a walk or even to a shop nearby unaccompanied by a male relative. Both students and trainers noted experiencing harassment from male drivers who would “look at [them] as if [they] were doing something wrong.” Such behaviors made students “feel uncomfortable” and explained therefore that “[they] do not go out or take a walk alone.”

Overprotectiveness was a barrier when women needed to go to a place far from where they lived, to join a hiking group, for example, or to practice a particular physical activity. Students' parents felt it was unsafe for their daughters to travel long distances alone. For example, Seba wanted to go horse riding, but her father refused to allow her to go to an equestrian club because of its distance:

I decided to join the equestrian club in the summer, but my parents refused and said, who will drive you there? And take you back? It was far away, and I cannot go far places with a strange driver, my parents are worried about my safety (Seba, student).

Participants also indicated that social perceptions of sport and physical activity in a male realm affected their ability to engage in physical activities. Mashael, a student spoke about her grandmother's belief that sport and physical activity “are forbidden and not allowed for females.” The responses from trainers also revealed that discrimination based on beliefs about gender roles extended to women going to the gym. Trainer Waad spoke of a client who joined her gym, but in the end, her husband would not allow it.

Before a month I had one woman told me that she wants to join a gym, but her community still sees it as an inappropriate thing for females to do… I had a woman who came to join the gym, but then her husband prevented her from doing so, she has her job and is responsible for her life, but her husband prevents her, no problem with money or time, or anything, it is all about the husband (Waad, trainer).

Some parents no longer subscribed to these gendered norms and were more supportive of their daughters' wishes to participate in sports. However, the social norm is still prevailing, and parents would often be subject to pressure from older members of their families who continued to subscribe to these discriminatory norms. Nuha, for example, was frustrated when she spoke of her uncle's continued criticism of her father allowing her to join a gym.

I think the social norms here negatively affected me. Oh, one of my uncles keeps criticizing my father saying, why do you let your daughters join a gym? It is more like telling him not to allow us to join it, he believes that sport is not for females (Nuha, student).

Trainers also noted that the social perception of women's physical activity was a matter of culture: “In a country where women's sports were neglected for a long time it was difficult to convince people about the importance of sports for women.” These were the words of Rasha, a trainer from Tunisia when we spoke about the differences between her country of origin, where she was able to get a degree in physical education, and Saudi Arabia regarding women's physical activity.

The final barrier was related to women's dress code in female-only places. Until recently, women in the Saudi community dressed strictly according to religious and cultural beliefs. This was based on religious ideology but in a practical sense hindered women from participating in gym activities. Trousers, for instance, were considered male clothing, so women had to wear long skirts and dresses instead. Although participants felt that social expectations on how women should dress were becoming more relaxed, traditional patterns could prevail within families and communities that were more conservative. Trainers described how some women wore skirts to the gym because of the ingrained belief “that it is forbidden for females to wear leggings.” Sometimes women wore skirts because their husbands only allowed them to go to the gym if they “wore skirts and not leggings.”

### Facilitators to Participation in Physical Activity

Two main themes were identified regarding facilitators to participation in physical activity. These related to factors such as personal motivations and family support. Personal motivators within the context of this study were factors that arose from the participant's internal desire to be active. These included noticing positive results from their physical activity, having company to workout with, and being concerned about their health. Family support encompassed family encouragement for women to either take up physical activities or maintain their commitment to their practice.

#### Personal Motivators Increased Levels of Physical Activity

Many students explained that seeing changes in their body shape, feeling less tired, and “feeling good” when they became “fit and healthier” motivated them to “do more and achieve more” in terms of physical activity. For Maryam, walking helped relieve stress and this was a motivator for engaging in physical activity.

When I feel stressed, I go out for a walk to reveal the stress and feel fresh again, I feel like I'm taking it all out and getting rid of everything (Maryam, student).

The importance of noticing tangible positive improvements in weight and shape and psychological wellbeing was echoed by trainers as reflected in Dalal's words when she spoke about women attending the gym.

When clients achieve something or notice a difference in themselves, this make them motivated to continue and when a client becomes stressed and can let it go in the class, she will come again because she thinks the gym is her release (Dalal, trainer).

Having someone to work out with whether a friend or family member was motivating to many students. This was especially true when first starting to exercise as they found it challenging “to start alone in the beginning.”

While body shape was a motivator for many, for others the main motivation was improving their overall health or following their doctor's advice to exercise. For example, Dana always intended to be active but did not commit until she was advised to practice cardiovascular exercise by her doctor as she experienced tachycardia: “When it came to my health I had to start.”

#### Family Support Encouraged Being More Physically Active

Some students explained that they were motivated either by seeing their mothers engaging in physical activity themselves or by accompanying them to the gym. For example, Mashael talked about how her mother loved walking, and that she always encouraged her to be active and to look after her body. The support from mothers was crucial in instances where male siblings openly criticized their sisters for joining a gym as portrayed below:

Support from family is important because at the time where my brothers were throwing comments, that did not affect me, because my mom and sisters kept encouraging me and say “No, keep it going” (Nuha, student).

Trainers also spoke of the role that mothers played in motivating their daughters, particularly when mothers exemplified the positive impacts of exercise.

A girl at the gym has told me, I decided to come to the gym after I saw my mom get ready every morning, I never imagined that I will see her waking up every day and be happy and active when she goes to the gym, I think she is addicted to it because she likes it (Heba, trainer).

It was clear that students who were encouraged by their parents to be active from an early age incorporated physical activity into their routine as part of their daily lives. In the words of Sara, who was studying linguistics and was a player on the University basketball team, seeing her father walk daily made her feel that she should be doing the same:

In the beginning, when we were kids, my dad loved being healthy, he used to take us with him to walk every time, so I grew up walking and moving a lot. I was not aware of the great benefits back then but now I do, and I think all the credits goes to my dad (Sara, student).

## Discussion

This article has provided important insights into the prevalence of physical activity among female University students in Saudi Arabia. In addition, in-depth interviews provided a unique opportunity for this study to further explore Saudi female's experiences and the barriers and facilitators to their engagement with physical activity. Among KSU female students in our study, 43% of participants were found to be physically active, while 29% were found to be inactive. These results support the work of Al-Drees, Abdulghani (50), who found that 45% of female medical students at the same University were active. However, that study specified only five types of physical activities—-walking, jogging, swimming, weightlifting, and football playing. The difference in domains of physical activity means results may be less congruent with our study as our research covered all domains of physical activity.

In contrast to the findings of this study, higher levels of inactivity have been reported in other studies conducted on Saudi adults (51). For instance, El-Sobkey (52) reported 83% inactivity levels, although the small sample size (100 participants) suggested this result may not have been entirely representative. Similarly, Albawardi et al. (53) reported 52% inactivity among women aged 18–58 years old. Reasons behind the variation in physical activity level between these and our study could be related to the differences in assessment methods, domains of activity, or study population characteristics. Albawardi et al. study, for instance, included females in office-based jobs, where this type of work was found to be associated with a reduction in physical activity (54, 55). Further, compared to our study, El-Sobkey's study included a wider age spectrum (10–70 years old), although research suggests that the ability to engage in physical activity declines with aging due to a decline in muscle mass and neuromuscular function (56, 57).

Consistent with the literature (35, 51), our study found that walking was the most common form of physical activity. Walking, however, is also a normal part of students' daily life, since they walk between colleges, to meet friends, and in shopping malls. Hence, it may be that the participants walked out of necessity and not necessarily as a preferred form of physical activity.

In-depth interview responses from participants highlighted the barriers and facilitators to female's participation in physical activity in Saudi Arabia. These identified barriers and facilitators in this study shared similarities with those of other contexts and populations, while also showing specific characteristics related to the Saudi context. The most common barriers stated by our study participants were environmental and structural factors, while the most common facilitators were personal factors. However, Saudi Arabia is a collectivist society like most of the Arab world where cultural norms and practices govern life (58). Hence, it is important to emphasize the relevance of socio-cultural context related factors mentioned by participants that may have influenced Saudi women's participation in physical activity. As Donnelly et al. (59) argue, women's and men's physical activity levels in a collectivist society are strongly influenced by social and cultural factors. Because studies addressing social and cultural factors within the Saudi community with regards to female's physical activity are scarce (18), such findings are important in understanding Saudi female's experiences and would be important in developing tailored interventions to improve women's physical activity level.

**Figure 1:**
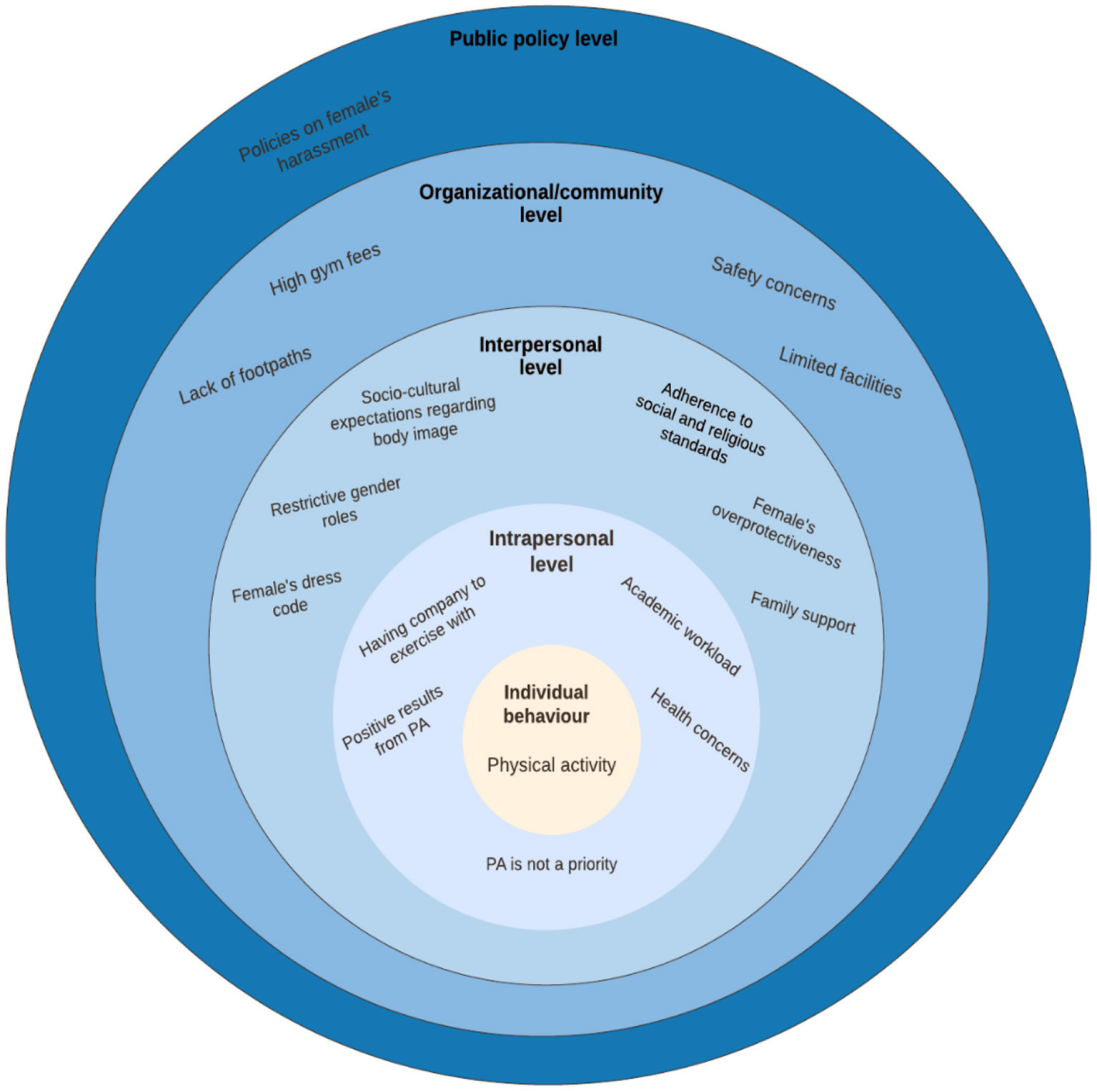
Social-ecological levels showing the barriers and motivators to females' engagement in physical activity.

We have chosen to interpret in the qualitative findings of this study through the Socioecological Model (SEM). The model includes concentric circles denoting the different layers of influence on human behavior (Figure 1) (60). At the core is individual behavior, in our case, this will be physical activity. Enlarging circles which also highlight the links between the different layers, show the different factors influencing individual behavior. These factors include *intrapersonal* (first layer) which relates to individual level factors, the second layer which is *interpersonal* (families, friends, social influence), the third layer *organizational* or *community level* (organizations and social institutions), and the outermost layer which is the *public policy* (national, state, local laws, and regulations) (61).

Viewed through this model, organizational factors were a salient contributor to students' participation in physical activity. This includes the environmental and structural factors and the social and cultural context as presented in the findings. Our study clearly showed that environmental and structural factors were key barriers. Safety was a concern to participants when it came to outdoor activities, mainly because of the lack of footpaths in the city. This finding supports those from Gordon-Larsen, Griffiths (62), Eyler, Baker (63), Omura, Hyde (64); and Odunitan-Wayas, Hamann (65) which were conducted in developed countries, suggesting that the built environment is a barrier common to several countries. Our study clearly shows there was less resources invested in facilities for women. Female-only gyms were scarce in Riyadh, especially when compared with the number of male-only gyms. This finding mirrored those of the previous studies conducted in Saudi Arabia, which have identified a lack of female-only gyms as a major impediment (12, 17, 18, 66–70). Comparing this finding with those from studies conducted worldwide confirmed that this barrier has negatively impacted female participation in physical activity (59, 64, 65, 71). However, it can be argued that the lack of female only gyms is not necessarily a barrier as working out in a gym is not the only means of physical activity. Alternatives to gym workouts such as swimming at aquatic centers or joining walking groups are available to women in Saudi Arabia. But these alternatives are not considered perhaps due to a lack of awareness. Moreover, environmental factors such as the weather and safety of the physical environment restricted our participants from walking. Overall, our findings have confirmed earlier research that has shown the impact of the physical environment on people's participation in physical activity. Consequently, to improve female's engagement in physical activity, there needs to be changes at an organizational level made to increase access to and safety of the physical environment.

At the organizational level of the SEM model, the social and cultural contextual factors also were a key deterrent for women to engage in physical activity. As can be seen in our study, culture has set the norms for what society expects of women and men. Gendered norms concerning what women should wear, how they should behave, and even their ideal body shape seems to play a significant role in restricting the extent to which women can be physically active. Our study clearly shows the Saudi dress code for women in public spaces and the societal restrictions on having female-only gyms was a significant barrier for women's engagement in physical activities. These findings are in agreement with Sharara and Akik (72), who concluded in their systematic review of studies from Arab countries that gender norms, especially the conservative dress code for women, hindered their participation in physical activity. Similarly, Amin and Suleman (73), reported that socially acceptable attire is a barrier to the participation of Saudi women in physical activities.

Control over women's dress in Saudi Arabia can be traced to the influence of the Wahhabi movement (74). Protecting women's virtues as perceived by the Wahhabi's were considered as a key element in the formation of a Muslim community; this translated to controlling the way women dressed and behaved in the public (74). As an example, women were expected to wear dresses and long skirts (at home as well as in female-only settings), but not trousers, which were seen as imitating men. These cultural norms on women's dress code is disappearing (75), especially with the Crown prince's push for more gender equality. In 2018, it was clarified by the Crown prince that women in Saudi Arabia should wear decent and respectful clothing, but that did not specify a black abaya or a blackhead cover (76). With such interventions the Saudi society appears to be becoming more relaxed in terms of women's dress code. Nevertheless, findings of this study strongly suggest such beliefs still prevail in some conservative families and some sectors of the community. Similarly, Donnelly et al. (59) found that the need to wear an abaya hindered Qatari women from participating in physical activity.

The recent changes to laws in Saudi Arabia pave the way for more gender equity. However, the literature shows changes of cultural and societal norms are deeply ingrained in the community, will be difficult and can take time (77–79). This is true for Saudi Arabia, where the societal and cultural norms have a religious basis, albeit at times misconstrued. Indeed, isolation and control of women by their male counterparts is to a large degree still apparent (80). This was evident in the last demographic survey in Saudi Arabia of illiteracy and education, which showed that 11 percent of the Saudi female population are illiterate, compared to three percent of males (81). Therefore, increasing public awareness of the importance of women's physical activity is likely to facilitate a change in socio-cultural norms and encourage behavioral change within the community.

In our study, cultural influence was also reflected in terms of males' overprotection of their female relatives. Gender conventions, such as women requiring to be chaperoned by a male family member in public, were noted by Sharara and Akik (72) as a barrier for women seeking to participate in physical activities. According to Gallagher and Maureen Searle (82), Saudi men saw their behavior toward women as protective rather than restrictive. This could be due to obsolete cultural norms that considered women as less capable humans who needed to be safeguarded by men (80). The empowerment of women to be physically active would be difficult unless we challenge cultural beliefs about women and challenge how these beliefs are perpetuated in society. On the other hand, given that Saudi Arabia is a collectivist community, the strength of the collectivist community could be harnessed to create a sociocultural environment that encourages, supports, and motivates people in Saudi Arabia to engage in physical activity.

Our study shows the complexity of the cultural context. One could argue that the need for protecting women would mean that women would not be harassed on the streets. However, our female participants felt unsafe to walk in their neighborhoods because they were harassed by male drivers with verbal abuse. Such harassment is likely due to some male factions of the society perceiving women should not be outside on the streets unaccompanied by a male relative and therefore they consider it is acceptable to harass females perceived to be breaking societal rules. At the same time such harassment experienced by females is likely to perpetuate the need for men to protect their female members of the family. The gendered norms relating to the expected behaviors of women clearly point to the predominant patriarchal culture (83) still prevailing in Saudi Arabia. The gendered norms are also perpetuated by the lack of laws and regulations that protect females in Saudi Arabia from harassment; up until 2018, there were no clear harassment deterrent laws or regulations in Saudi Arabia (84, 85). Although *public policies*, which is at the outer level of the SEM were not directly elicited in the interviews, the findings provide clear evidence for the importance of public policies, to increase the levels of physical activity among women.

The intrapersonal factors depicted in the SEM model is reflected in our study as *personal* factors were clearly illustrated in the findings of this study. While the organizational level factors were barriers to physical activity, the intrapersonal factors in our study were facilitators. Positive improvements in body shape, measurements, or weight motivated our participants to maintain their activity. Previous studies have shown that improved body shape, weight control, and feeling good from exercising were motivators for their participants to engage in physical activity, which supports our findings (69, 86–91). Also, health scares as a motivator of becoming active have been observed in earlier studies (59, 68, 69, 86, 88–90). Hence, promoting awareness of the benefits of physical activity to health may have a positive effect on encouraging females to participate in physical activity.

Further, consistent with the literature (59, 63, 86–90, 92), the findings from this study point to the significance of the interpersonal factors of the SEM model. Social and family support motivated participants to engage in physical activity with support from parents being the predominant motivator. This may partly be due to the teaching of Islam, which emphasizes respect and obedience to parents (93). It could also relate to the fact that family is a central pillar of Saudi society which forms the individual's social circle, particularly for women (94). Family involvement should therefore be considered when developing intervention programs aiming at improving females' participation. The involvement of families, according to Al-Kaabi et al. (95) could potentially enhance adherence to physical activity and the continuity of it.

## Strengths and Limitations

The main strength of this study was the use of qualitative methods to expand on the findings of the quantitative survey to better understand women's experiences of undertaking physical activity in Saudi Arabia. The qualitative component provided an in-depth understanding of the barriers and facilitators leading to the identification of factors not reported in previous studies which solely relied on quantitative data collection methods. The research design, data collection, analysis, and writing up of findings, have adhered to the COREQ guidelines for qualitative research (45).

The findings of this research are subject to some limitations. One limitation was the use of a self-administrated survey (IPAQ), which had the potential to over-or under-report the level of activity due to recall and/or social desirability biases (96). About two-thirds of the data were excluded which might have affected the representativeness of female University students. There was also a possibility of sampling bias since it would have been more likely that physically active students would have taken the survey than less physically active students. Furthermore, since there was no demographic data collected from a large number of students (about 70%) who opened the survey link but did not go beyond the first question meaning that no reason was given for them not completing the survey, it is impossible to comment on whether these participants differed from those who completed the survey. Moreover, the questionnaire design, while allowing assessment of physical activity levels, could not further explore possible facilitators and barriers to compare with the qualitative findings. Finally, since the study was limited to a relatively privileged group of female University students and female gym trainers, the findings cannot be generalized to the entire Saudi female population.

## Recommendations

Roads in Saudi Arabia are primarily constructed to meet the needs of motor vehicle users. While some efforts have been made to encourage walking and cycling in specific neighborhoods through the creation of walkways and parks, no clear policies or plans to support alternate modes of transportation on a broader scale are found (97). As a result, clear rules and plans for alternative transportation infrastructure are required as the foundation for a sustainable and liveable environment in which people may simply walk or cycle. To reduce climate impacts on pedestrians, sidewalk greening to provide shade, as well as water coolers (sprays) are recommended in Riyadh to reduce urban heat and dry wind in the city.

Public policies relating to accessing venues where females can safely engage in physical activity can help in creating a conducive environment in Saudi Arabia. Therefore, the establishment of female-only gyms with affordable membership costs that are equitably located across the country are recommended to support female's engagement in physical activity.

The active engagement of different stakeholders when planning interventions or programmes to encourage physical activity in the female population is also recommended. Public health practitioners, politicians, gym owners, trainers, the education sector, and women from the general community could all be involved. The range of stakeholders should reflect the diversity of the Saudi population, especially across different generations, of traditions and customs and levels of openness to change. Awareness of these contextual factors can help to identify the best approach to improving the physical activity of the targeted population group.

Future research directions can involve recruiting different population groups, such as elderly women, adolescent girls, and regional populations. These studies can be extremely beneficial, especially in light of the social changes that are taking place in Saudi Arabia as a result of Vision 2030. Community perceptions of women's engaging in physical activities can also be explored and the impacts of social change on their participation evaluated.

## Conclusion

In conclusion, this study contributes to a better understanding of women's experiences of engaging in physical activities within the cultural norms and social practices in Saudi Arabia. This study adds to the evidence that physical activity is a contextualized experience that is shaped by the society, culture, and background of the study group. In view of recent developments in women's rights in Saudi Arabia, these findings make a significant contribution to current knowledge. It is hoped that knowledge gained from this study might provide a basis for organizations and public health authorities to better tailor physical activity interventions to address the needs and perceptions of women.

## Data Availability Statement

The original contributions presented in the study are included in the article/Supplementary Material, further inquiries can be directed to the corresponding author/s.

## Author Contributions

NA: study conception and design, data collection, data analysis, and drafting the manuscript. HR: study conception and design, acquisition of qualitative data, contributed to the analysis of qualitative data, contributed to early drafts of the manuscript, and assisted in revising it for intellectual content. AB-B: study conception and design, acquisition qualitative data, contributed to the analysis of qualitative data, and assisted in revising it for intellectual content. JR: analysis of qualitative data, contributed to early drafts of the manuscript, and assisted in revising it for intellectual content. TV: study conception and design, acquisition quantitative data, contributed to the analysis of quantitative data, and assisted in revising it for intellectual content. GK: contributed to the analysis of qualitative data and assisted in revising it for intellectual content. All authors contributed to the article and approved the submitted version.

## Conflict of Interest

The authors declare that the research was conducted in the absence of any commercial or financial relationships that could be construed as a potential conflict of interest.

## Publisher's Note

All claims expressed in this article are solely those of the authors and do not necessarily represent those of their affiliated organizations, or those of the publisher, the editors and the reviewers. Any product that may be evaluated in this article, or claim that may be made by its manufacturer, is not guaranteed or endorsed by the publisher.
