# Supplementary material for: Exploring Female University Students' Participation in Physical Activity in Saudi Arabia: A Mixed-Methods Study
Source: Front Public Health. 2022 Mar 18;10:829296. doi: 10.3389/fpubh.2022.829296 (PMC8971611; doi:10.3389/fpubh.2022.829296)
Supplement: Supplementary file 1 [file Table_1.docx]

# Supplementary material

**Interview guide for KSU students**

1. Tell me about a typical week in your life. Probe:
   - - - What activities do you consider part of your routine?
2. How easy is it as a woman, to be physically active in Saudi Arabia? Probe:

What makes it easy?

What makes it difficult?

1. In your opinion, what might influence women’s motivation to engage in PA? probe:
   - - - What might encourage them?
       - What might discourage them?
2. What challenges do you think women in Saudi Arabia might face when they want to engage in PA? probe:
   - - - Why would they face these challenges?
       - How should they deal with it?
3. How do you feel about your activity level? Probe:
   - - - What contributed to this feeling?
4. If you decided to engage in PA, what options are available for you?
5. Tell me about your previous experiences in trying to engage in PA. Probe:
   1. In case there are previous experiences:
      - - Why did you decide to engage in PA?
        - What was the most satisfying part of your experience?
        - What was the most dissatisfying part of your experience?
   2. In case of no previous experiences:
      - - What are the reasons that caused you not to engage in PA?
6. What would motivate you to engage in activity/ or maintain your activity level?
7. What barriers do you think you will face (or faced) when you decide to engage in PA? probe:
   - - - Were you able to overcome them? Why and why not?
       - What would you do (or did) to overcome them?
8. In your opinion, how do does the surrounding environment in your life influence your activity level? Probe:
   - - - How would your family influence your activity?
       - How would your friends influence your activity?
       - In what ways does the community influence your activity level (social and cultural norms)?

**Interview guide for female exercise trainers**

1. In general, how do you see female’s PA in Saudi Arabia? Probe:
   - - - Why?
       - What contributed to your view?
2. How easy is it for females in Saudi Arabia to be active? Probe:
   - - - What makes it easy?
       - What makes it hard? (Reasons why women might not engage in PA)
3. In your opinion, what factors might influence the activity level of females in Saudi Arabia? Probe:
   - - - What factors might positively influence female’s activity?
       - What factors might negatively influence female’s activity?
       - Influence of social and cultural norms in the Saudi community.
       - Family influence
4. What barriers do you think might hinder females from engaging in PA in SA? Probe:

- How would these act as barriers?

1. How can we encourage/help females to become more active? Probe:
   - - - Who should be included to help in improving their activity level?
       - What role community could play to improve female’s activity levels?
2. Can you talk about your experience with women trying to improve their activity?
   - - - What are the main goals women are aiming for when they want to improve their activity?
       - What approaches were more effective and helped females to engage in PA? Why?
       - What might negatively influence their commitment?
       - What might motivate them to continue?

# Data collection method

As little is known about women’s experiences of engaging in physical activity in Saudi Arabia, it was necessary to use methods that would generate valuable insight into their experiences and beliefs, including the challenges and facilitators they encountered in their engagement with physical activity. Thus, a qualitative approach was chosen for this part of the first phase of the study as it would make possible in-depth understanding and exploration of the interviewees’ perceptions based on their experiences and beliefs (1). By using face-to-face in-depth interviews to collect such data, we hoped to gain insightful knowledge of women’s perspectives regarding the topic (2, 3). The interviews were designed to be flexible and interactive in style to inform a deeper understanding of participants’ situations in their natural setting (4). Moreover, this approach would allow participants to explore the topic freely and helped in collecting rich information about participants’ perspectives and beliefs (5).

Semi-structured interview guide was used that included a list of questions and topics that were covered during the interviews (6). This allowed interviewees’ experiences to unfold in an open discussion that would turn to whatever relevant issues respondents raised or experiences they wanted to reveal (6, 7). In the interviews with students, the main areas of discussion included a) their experiences regarding physical activity, including the barriers and facilitators they perceived to influence their physical activity participation; b) their expectations and needs in participating in physical activity; and c) what approaches they might propose to improve their physical activity levels. In the interviews with the key informants, the main areas included a) their views about the physical activity status of women in Saudi Arabia; b) their experiences with women attempting to participate in physical activity; and c) their recommendations for improving the physical activity levels of women in Saudi Arabia.

Interviews were conducted in vernacular Arabic, although invitations and consents were written in formal Arabic. Formal Arabic is not used in daily communication and, moreover, informal Arabic allowed respondents to talk and express their views and feelings fluently. Respondents were also free to speak in either Arabic or English; some preferred to move between the two languages to clarify or better explain their points.

The interviews started with general and simple questions to establish rapport with the interviewees and encourage their confidence, which helped to draw rich data from our conversation (2). A two-way interaction was invited that allowed respondents to ask questions if they wanted to, thus building our rapport (8). During the interviews, NJ frequently summarised what the participants said, and checked with each participant that she had correctly interpreted and reflected on her words. Due to practical difficulties, it was not possible to contact all the participants to discuss the findings from the interviews and obtain their feedback.

All interviews with students and key informants were conducted and recorded in Arabic after permission had been sought. As interviews were conducted in Arabic, translation into English was required, which could misconstrue speakers’ linguistic constructions or meaning (9). The recommendation that a fluid description of the meaning be used instead of a word-by-word translation was followed to retain the original meaning (9). Also, to reduce the chances of the loss of meaning, van Nes, Abma (9) recommend that researchers use the original language for as long as possible during the analysis. So, to address these issues NJ transcribed all the interviews in Arabic (a native Arabic speaker and speak and write English well) and began the analysis of the Arabic transcripts manually before translating them into English. Then two of the English interviews were back-translated into Arabic by a second person who has a bachelor’s degree in linguistics and is fluent in both languages. The back-translated interviews were reviewed by NJ while listening to the recording, to ensure that translation was accurate, and the meaning-transfer-chain was not lost during translation (9). After interviews were translated into English, NJ analysed them in NVivo 12 (10). Codes from both the Arabic and English transcripts were compared to check if they were similar (9).

The research process was documented in the field journal, the minutes of meetings with the authors, reflective notes taken after each interview, and notes of meetings with authors to discuss the field work. During the coding process, codes were revised and adjusted several times, and all the steps of the analysis were documented in the journal so as to keep track of any changes and the reasons for making them. During the analysis and to eliminate any possibilities of mistakes the data were re-checked, literature was searched for explanations of the findings, and discussions with the authors were held to discuss the analysis.

Thematic analysis was used to analyse the data derived from the interviews (11). The analysis was guided by the six steps advised by Braun and Clarke (11) for analysing data. As the analysis requires immersion in the data, NJ started by familiarising herself with the data (11), by first listening to the interviews and transcribing them into Arabic. NJ was then able to summarise each interview and record her reflections on each of them. NJ started coding the Arabic interviews manually, since NVivo does not support the Arabic language. Then, interviews were translated into English and uploaded into NVivo. After that, NJ read the English interviews, and while re-reading them and simultaneously listening to the recording, I noted any thoughts or ideas that came to my mind. Then, initial codes were generated in NVivo, then subsequently adding codes that seemed to be related to each other by theme.

After sorting all codes under subthemes and themes, NJ continued reviewing, moving some codes under another theme, and merging others into one code. At that point themes were more descriptive than analytic, so they were left for two weeks to review the coding with fresh eyes, trying to go beyond thematic description to a systematic analysis. This process took around three weeks and resulted in major changes to the themes. At the same time, GK (a native Arabic speaker with a PhD in public health) coded two interviews separately. Then NJ and GK compared their analyses and discussing the codes and themes at length resulted in further modifications to them.

Themes were then reviewed to check if they related to the coded data. This was done in regular meetings with all authors where we discussed and reviewed each code and example quotations. This process was necessary to refine our themes and to generate clear definitions for them so they would illustrate the larger story. The findings were reported in a way that referenced and related to the research aim and objectives.

**References**

1. Mack N, Woodsong C, MacQueen KM, Guest G, Namey E. Qualitative research methods: a data collectors field guide. 2005.

2. Gill P, Stewart K, Treasure E, Chadwick B. Methods of data collection in qualitative research: interviews and focus groups. British Dental Journal. 2008;204(6):291-5.

3. Creswell JW. Research design: qualitative, quantitative, and mixed methods approaches. 4th ed. 2014;Thousand Oaks, California: SAGE Publications.

4. Legard R, Keegan J, Ward K. In-depth interviews. Qualitative research practice: A guide for social science students and researchers. 2003:138-69.

5. Ritchie J, Lewis J. Qualitative research practice: A guide for social science students and researchers: Sage publications; 2003.

6. Galletta A. Mastering the semi-structured interview and beyond: From research design to analysis and publication: NYU Press; 2013.

7. Ritchie J. Not everything can be reduced to numbers. Health research. 2001:149-73.

8. Braun V, Clarke V. What can “thematic analysis” offer health and wellbeing researchers? International Journal of Qualitative Studies on Health and Well-being. 2014;9(1):26152.

9. van Nes F, Abma T, Jonsson H, Deeg D. Language differences in qualitative research: is meaning lost in translation? Eur J Ageing. 2010;7(4):313-6.

10. QSR International Pty Ltd. NVivo (Version 12). 2018.

11. Braun V, Clarke V. Using thematic analysis in psychology. Qualitative Research in Psychology. 2006;3(2):77-101.
